# Prefrontal Cortex and Social Cognition in Mouse and Man

**DOI:** 10.3389/fpsyg.2015.01805

**Published:** 2015-11-26

**Authors:** Lucy K. Bicks, Hiroyuki Koike, Schahram Akbarian, Hirofumi Morishita

**Affiliations:** ^1^Department of Psychiatry, Icahn School of Medicine at Mount Sinai, New YorkNY, USA; ^2^Department of Neuroscience, Icahn School of Medicine at Mount Sinai, New YorkNY, USA; ^3^Department of Ophthalmology, Icahn School of Medicine at Mount Sinai, New YorkNY, USA; ^4^Mindich Child Health and Development Institute, Icahn School of Medicine at Mount Sinai, New YorkNY, USA; ^5^Friedman Brain Institute, Icahn School of Medicine at Mount Sinai, New YorkNY, USA

**Keywords:** social cognition, social behavior, prefrontal cortex, autism, schizophrenia

## Abstract

Social cognition is a complex process that requires the integration of a wide variety of behaviors, including salience, reward-seeking, motivation, knowledge of self and others, and flexibly adjusting behavior in social groups. Not surprisingly, social cognition represents a sensitive domain commonly disrupted in the pathology of a variety of psychiatric disorders including Autism Spectrum Disorder (ASD) and Schizophrenia (SCZ). Here, we discuss convergent research from animal models to human disease that implicates the prefrontal cortex (PFC) as a key regulator in social cognition, suggesting that disruptions in prefrontal microcircuitry play an essential role in the pathophysiology of psychiatric disorders with shared social deficits. We take a translational perspective of social cognition, and review three key behaviors that are essential to normal social processing in rodents and humans, including social motivation, social recognition, and dominance hierarchy. A shared prefrontal circuitry may underlie these behaviors. Social cognition deficits in animal models of neurodevelopmental disorders like ASD and SCZ have been linked to an altered balance of excitation and inhibition (E/I ratio) within the cortex generally, and PFC specifically. A clear picture of the mechanisms by which altered E/I ratio in the PFC might lead to disruptions of social cognition across a variety of behaviors is not well understood. Future studies should explore how disrupted developmental trajectory of prefrontal microcircuitry could lead to altered E/I balance and subsequent deficits in the social domain.

## Introduction

Social behavior deficits are a fundamental dimension of many psychiatric disorders including the neuordevelopmental disorders ASD and SCZ, yet much remains to be learned about the underlying pathophysiology of these deficits. In 2010, the NIMH put forward a Research Domain Criteria^1^ initiative, which establishes a framework aimed at encouraging researchers to investigate common behavioral domains and neurobiological mechanisms that underlie multiple disorders. This collaborative effort identified five major domains that are disrupted across psychiatric disorders including cognitive systems, negative valence systems, positive valence systems, arousal/regulatory systems, and last but not least, social processing^1^. While defects in social processing underlie multiple disorders, it is still unclear if a common neurobiology mediates a ‘social brain.’ The prefrontal cortex (PFC) may be a candidate regulator in mediating social cognition (see **Table 1**) in both humans and rodents. In humans, social cognition develops throughout childhood and adolescence, and the appropriate maturation of the circuitry within PFC may play a key role in this trajectory. However, more detailed insights into the underlying molecular and cellular mechanisms can only be acquired by the study of small laboratory animals. Here we discuss the role of the PFC in mediating a broad range of social behaviors in rodents, with the hope that this framework might provide valuable insights for evaluating animal models of human psychiatric disease.

**Table 1:** Glossary.

**Social cognition** – The set of mental operations used to identify and interpret social signals and the use of those signals to guide behavior. We use this term in a broad sense, to incorporate social behaviors including social motivation, and group related behaviors including dominance and hierarchy.
**Social motivation** – An intervening variable that describes the desire of an organism to seek out social contact and interaction with conspecifics. Experimental procedures examining social motivation often use dependent variables of social approach, social investigation, and social contact, all of which are aspects of a more general ‘social motivation.’
**Social memory** – The ability to recognize other individuals that have been previously encountered.
**Social hierarchy** – The establishment of dominant and subordinate relationships between animals living in groups. These relationships often relate to aggressive behavior and access to resources including food and mates. However, establishment of hierarchies also includes species-specific behaviors not related to aggression.
**Sociability** – A tendency or trait describing the degree of social motivation.
**E/I balance** – This concept describes the ratio of cellular excitation to inhibition, usually within the cortex.


## Social Cognition In Human: Relevance To Psychiatric Disorders

**FIGURE 1:**
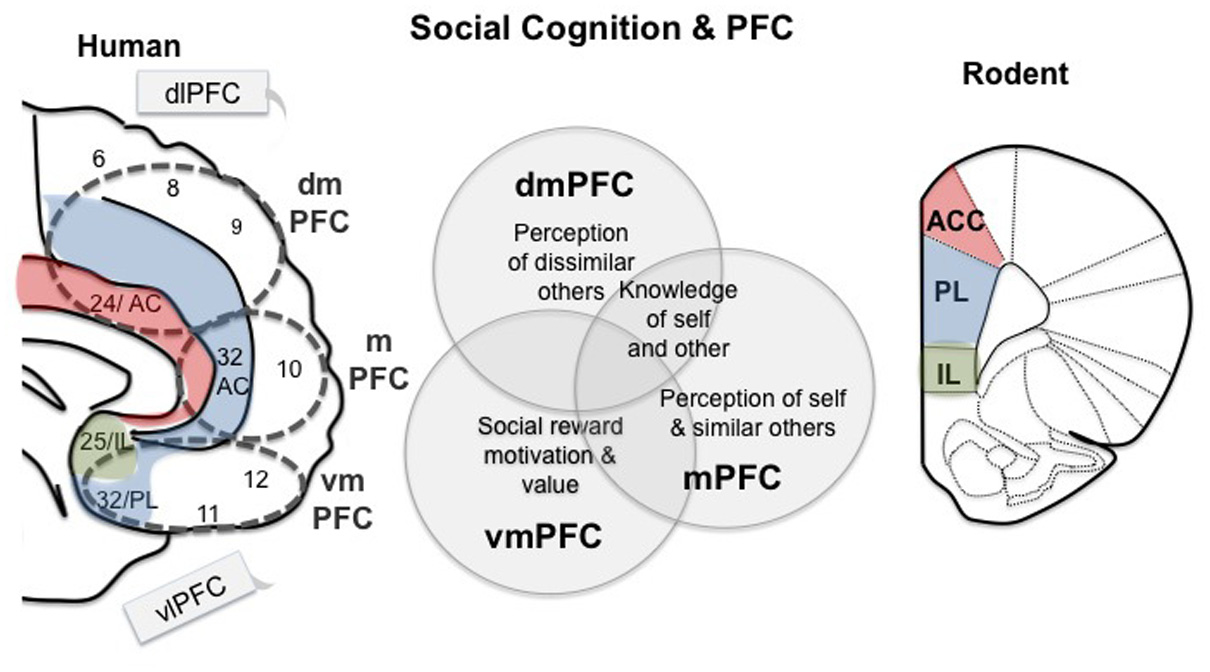
**Working model for prefrontal regions involved in social cognition in human and mouse.** Medial regions of the prefrontal cortex (PFC) are specifically related to social behavior, while the lateral regions, dlPFC and vlPFC, are sometimes active during social tasks, but are considered ‘domain general.’ The dmPFC is involved in perceptions of others as well as cooperation (Amodio and Frith, 2006; Mitchell et al., 2006). The mPFC has also been associated with perceptions of others, but some research suggests that it is more strongly associated with perceptions of self and similar others (Johnson et al., 2002; Mitchell et al., 2006; Mitchell, 2009). Ventral regions of the PFC are involved in social reward and punishment, motivation and ‘value’ (including economic) (de Quervain et al., 2004; Fehr and Camerer, 2007; Kohls et al., 2012). Parts of these divisions in the human brain share homology with the rodent PFC, as indicated. VmPFC contains BA 25, which is homologous to the rodent IL region, and area 32 is homologous to the PL. Area 24 in humans shares homology with the rodent ACC. These regions thus may play a shared role in social cognition across mammalian lineages.

Social cognition can be broadly defined as the set of mental operations used to identify and interpret social signals, and the use of those signals to guide the flexible performance of appropriate social behaviors given a changing context (Millan and Bales, 2013). In this review, we focus on three major facets of social cognition: social motivation, knowledge of self and other, and group dynamics, because these aspects of social behavior have shown relevance to psychiatric disorders, not only in humans but also in translational animal models (**Figure 1**). It should be noted that these three aspects of social cognition are not necessarily mutually exclusive (Ochsner, 2008; Green et al., 2015). We feel focusing on these three behaviors allows for an interesting comparison between social cognition in humans and animal models.

Social motivation, or the desire to seek social contact, is an elemental social behavior that includes social orienting and approach, social reward and cooperation, and maintaining social contacts (Chevallier et al., 2012). Behaviors such as social affiliation, orienting, and approach are evolutionarily conserved behaviors that are present in many species, including some invertebrates (Insel and Young, 2000; Toth and Robinson, 2007; Rosa Salva et al., 2011; Sheehan and Tibbetts, 2011). Social motivation also emerges early in development (Di Giorgio et al., 2012; Jakobsen et al., 2015). For example, newborns prefer to look at faces with open eyes, showing a natural propensity for social interaction from birth (Farroni et al., 2006). Social motivation is disrupted in many psychiatric disorders, including ASD and SCZ (Dawson et al., 1998; Buchanan, 2007; Chevallier et al., 2012; Blanchard et al., 2015; Dubey et al., 2015; Fervaha et al., 2015). The social motivation theory of autism suggests lack of social interest in childhood may contribute to additional social cognitive deficits that emerge later in development (Chevallier et al., 2012), suggesting the possibility that social motivation is a developmental and evolutionary building block required for other social behaviors.

Knowledge of self and others is an essential element of human social cognition. This level includes behaviors like facial recognition, empathy, evaluating emotion and motivation of others [‘mentalizing’ also known as theory of mind (ToM)], knowledge about the affective state and personality traits of the self and others, implicit and explicit biases, and moral judgments. Behaviors in this category rely on a human ability to use knowledge about ones own mental state to make inferences about the mental states of others (Mitchell, 2009). Many social behaviors require both motivation and knowledge of self and others, like making charitable contributions and engaging in cooperation. Other behaviors, like perspective taking and moral judgments are somewhat independent from social motivation. Behaviors related to knowledge of self and others are disrupted in a variety of psychiatric disorders including ASD and SCZ (Perner et al., 1989; Frith, 1994; Corcoran, 2001; Senju, 2012). For example, the majority of children with ASD do not pass the false-belief test, a common ToM test that examines the ability of subjects to recognize that others have differing sets of knowledge about a scenario depending on what they see (Baron-Cohen et al., 1985).

Group living is common in mammalian societies, and the evolutionary pressure to adapt to living in groups has been proposed as a main driver of the evolution of the primate PFC (Dunbar and Shultz, 2007; Adolphs, 2009; Dunbar, 2009). Studies examining group living are concerned with the interaction between the individual and the social group as well as the emergent properties of the group as a whole. Dominance hierarchies are particularly well established in many different species, and this complex group behavior involves many other important social behaviors, like knowledge of the position of the self relative to others in the group, communication, and social decision making. Additionally, hierarchies have important consequences for the health and well being of individuals within the group, making them an interesting behavior for further study. For example, in non-human primates, subordinate status within a social hierarchy can be a potent stressor, and in humans health is strongly influenced by socioeconomic status (Sapolsky, 2004). In addition to hierarchies, some social phenomena that emerge from group living in humans are organization of governments, societies, and cultures.

## PFC Regulation Of Social Cognition In Human Health And Disease

The PFC has been implicated in a wide range of behaviors including working memory, decision making, goal-directed behaviors, and social behavior (Duncan and Owen, 2000; Wood and Grafman, 2003; Wood et al., 2003). The PFC is connected with other cortical and sub-cortical regions of the brain, including hub regions of ‘the social brain’ such as the nucleus accumbens (NAc), amygdala, ventral tegmental area (VTA), hypothalamus, and regions of the cortex involved in processing sensory and motor inputs and responses (Ongur and Price, 2000; Croxson et al., 2005; Wise, 2008). Additionally, regions of the PFC are densely interconnected (Passingham et al., 2002).

Social behaviors within all three levels of social cognition are subserved by the PFC acting in conjunction with other cortical and subcortical regions (**Figure 1**). However, different regions within the PFC are associated with different categories of social cognition (Wood, 2003; Wood and Grafman, 2003; Amodio and Frith, 2006; Mitchell, 2009). The primary brain regions that underlie social motivation are regions that are related to reward behaviors in general, including the ventral striatum, amygdala, and the ventromedial prefrontal cortex (vmPFC: Brodmann cytoarchitectonic areas (BA) 25, 32 11,12 and parts of 10) (Kas et al., 2014) which includes the medial orbital frontal cortex mOFC (BA 11 and parts of 10) (Chevallier et al., 2012) and the perigenual anterior cingulate cortex (ACC: BA 25 and parts of 32) (Meyer-Lindenberg and Tost, 2012) (**Figure 1**). Many lines of evidence have demonstrated the importance of the vmPFC for social motivation and reward. For example, patients with vmPFC lesions demonstrate social isolation and apathy (Barrash et al., 2000) and decreased prosocial behavior in several social decision making games (Krajbich et al., 2009). Additionally, subjects who rate highly on a psychopathy scale show a decrease in the activity of the vmPFC when choosing to cooperate compared with controls (Rilling et al., 2007). The vmPFC is engaged when subjects feel social acceptance (Moor et al., 2010), and is activated when learning which cues predict social reward (Lin et al., 2012). Interestingly, performance on a vmPFC dependent task in ASD patients ages 3–4 correlates with joint-attention ability, suggesting a relationship between social motivation deficits and vmPFC functioning in early ASD development (Dawson et al., 2002b). Children with ASD show decreased vmPFC and striatal responses to social reward in an implict learning task (Scott-Van Zeeland et al., 2010). Interestingly, children with ASD also show decreased responses to peer rejection in regions of the vmPFC and vlPFC (Masten et al., 2011), including in the subgenual ACC. These findings show impaired vmPFC responses to social reward and rejection in ASD patients, which are closely related to social motivation. Regions of the subgenual ACC (BA 32 and 24) have been implicated in social motivation in primates as well. For example, lesions of the ACC gyrus (BA 32 and 24) disrupt social interest and valuation in macaques (Rudebeck et al., 2006; Noonan et al., 2010).

Social behaviors requiring knowledge of self and other are consistently related to activation within the PFC, and in particular a medial region of the PFC that includes the mPFC and the dmPFC (Amodio and Frith, 2006) (**Figure 1**). This area is activated by a diverse range of social cognitive tasks that include evaluating one’s own mental state or determining whether certain personality traits apply to you, perception and judgment of the mental states of others (ToM), moral decision making, cooperation, and empathizing about the pain of others (Amodio and Frith, 2006). In healthy adults, this region is typically more active in joint attention tasks than in solo attention tasks, but this difference does not exist in adults with ASD (Redcay et al., 2013). In ASD patients, alterations in mPFC activity and connectivity are a consistent finding, and these deficits likely relate to social deficits in this disorder. For example, decreased blood flow in the mPFC in children with ASD correlates with poor social functioning (Ohnishi et al., 2000). ASD patients performing a ToM task that involves attributing mental states to geometric figures show decreased activity within the mPFC relative to controls (Castelli et al., 2002; Kana et al., 2015) and decreased functional connectivity between mPFC and parietal regions (Kana et al., 2009). SCZ patients and their unaffected relatives show impaired performance on ToM tasks and decreased mPFC (Mohnke et al., 2015) and inferior frontal gyrus (Das et al., 2012) activation while performing this task. These findings demonstrate a common mPFC hypoactivation in behaviors related to knowledge of self and other in ASD and SCZ. Since ASD and SCZ both share neurodevelopmental origins, it is important to examine the development of these deficits in social processing. The mPFC is responsive to social stimuli in developing infants (Grossmann, 2015). In particular, the mPFC is sensitive to signs that an interaction is directed at the infant (‘self relevance’) (Grossmann, 2013). For example viewing a mothers smile, or hearing infant directed speech activates this region (Saito et al., 2007; Minagawa-Kawai et al., 2009). Additionally, the mPFC is engaged in joint engagement tasks in infants, in which an adult uses gaze to direct the attention of an infant to a third object (triadic interaction) as well as during a dyadic mother–infant social interaction (Urakawa et al., 2014; Grossmann, 2015). Joint engagement tasks and gaze following rely on both social motivation and interpretation of social signals, and are some of the earliest behavioral predictors of ASD (Toth et al., 2006). These findings suggest that some of the same brain regions may underlie knowledge about self and other throughout development. The mPFC shows decreased glucose metabolism in a population of Romanian orphans that show social and cognitive impairments, suggesting this region is sensitive to early life stressors that result in social deficts (Chugani et al., 2001). Interestingly, patients who sustained damage to their mPFC during infancy demonstrated anti-social behavior and poor moral decision making in adulthood, in contrast to patients who sustained damage to this region as adults (Anderson et al., 1999). This finding suggests that this region may have a developmental critical period for establishing an appropriate social cognition in humans.

Within the category of knowledge of self and other, attempts have been made to dissociate contributions of different brain regions. For example, emotional/implicit social cognition has been contrasted with explicit or effortful social cognition. Regions outside of the PFC including the inferior frontal gyrus and amygdala are primarily associated with the former, and dmPFC and mPFC are primarily associated with the later (Frith and Frith, 2008; Mitchell, 2009; Shamay-Tsoory et al., 2009). Within the PFC, many theories dissociate contributions of lateral PFC with medial PFC. Some research suggests that lateral PFC regions are ‘domain general’ and are recruited to resolve conflicts in social cues while medial PFC regions are specific to the use of contextual social cues to guide social behaviors like joint-attention, social reward, moral judgments and mentalizing (Wood, 2003; Wood and Grafman, 2003; Amodio and Frith, 2006; Zaki et al., 2010). An alternative theory to explain medial and lateral PFC contributions to social cognition posits that the mPFC is involved in tasks that require internal social processing of both self and other, for example empathy, mentalizing, self-reflection and personal moral reasoning whereas the lateral PFC is part of a network that is activated by externally guided processing in the social domain, for example imitation, abstract social reasoning, and resolving conflict in social cues (Lieberman, 2007). In psychiatric diseases that share social deficits, lateral regions of the PFC have also been associated with poor social functioning. For example, activation in the dorsolateral PFC (dlPFC: BA 9, 46: **Figure 1**) in response to social cues is aberrant in patients with SCZ (Shin et al., 2015). Interestingly, transcranial direct simulation of the dlPFC improved some parameters of social cognition, such as ‘emotion identification,’ in subjects with SCZ (Rassovsky et al., 2015). Additionally, both paranoid SCZ and ASD patients show decreased activation in the ventrolateral PFC (vlPFC: BA 47, 45, 44: **Figure 1**) when making trustworthiness judgments (Pinkham et al., 2008). Finally, some research dissociates functions of the dorsal and medial regions of the PFC, suggesting the dmPFC is engaged when mentalizing about others, while the mPFC is engaged preferentially in self-referential tasks like preference and affective state judgments (Gusnard et al., 2001; Johnson et al., 2002) as well when taking the perspective of similar, but not dissimilar others (Mitchell et al., 2006). While no theory provides a conclusive description of the contributions of sub-regions of the PFC to different aspects of knowledge about self and other, there is a consensus that both the dmPFC and mPFC are specifically related to this form of social cognition.

The third category of social cognition, group dynamics, relies on both motivation and knowledge of self and other. Living in groups often involves a hierarchical organization, and this organization requires that individuals perceive both their own status within the group, as well as the status of others around them in order to behave appropriately (Rowell, 1974; Watanabe and Yamamoto, 2015). The neural mechanisms supporting perception of hierarchy in humans rely largely on the PFC acting in conjunction with subcortical regions including the amygdala and ventral striatum, which help interpret the stressful or rewarding values often associated with changes in status (Wang et al., 2014; Watanabe and Yamamoto, 2015). In macaques, gray matter volume in the rostral and dorsal PFC correlates with the size of a social network and with social status (Sallet et al., 2011; Noonan et al., 2014) and in humans the gray matter volume of the vmPFC varies with both metalizing competence and social network size, showing a shared neural circuit for distinct facets of social cognition (Lewis et al., 2011). Patients with lesions spanning vmPFC and mPFC do not alter their behavior according to differing ranks in a professional setting, suggesting disruptions in this region lead to poor understanding of the social cues dictated by hierarchy (Karafin et al., 2004). The lateral PFC has also been shown to have an important role in perceptions of hierarchy. For example, viewing an individual that ranks above you in a hierarchy activates the dlPFC in both stable and unstable hierarchy conditions, and activates the mPFC and the amygdala only in unstable hierarchy conditions (Zink et al., 2008). This suggests that the lateral regions of the PFC may be important for knowledge about your own place in a hierarchy, while activity in the mPFC and amygdala may help coordinate appropriate behaviors when a hierarchy is changing, and knowledge must be continually updated.

Overall, there is strong evidence in the field of social cognition that medial regions of the PFC including the mPFC, dmPFC, and the vmPFC are crucial for a wide variety of behaviors that include motivation, understanding of the self and others, and formation of complex group behaviors (**Figure 1**). In the following, we compare the human data presented above with rodent models that converge on the hypothesis that evolutionarily shared regions of the mPFC mediate social behavior across species.

## Social Cognition And The PFC In Rodents

If animal models are to provide useful insights in evaluating how alterations within the PFC circuitry can lead to social deficits in models of human disease, we must first determine to what extent ‘social cognition’ is related to a consistent neural mechanism across mammalian lineages, including rodents. Some controversy exists in translational neuroscience about the existence of the rodent PFC, and many researchers have debated the homology between specific regions in primate and rodent forebrain (Preuss, 1995; Uylings et al., 2003; Wise, 2008). A consensus has emerged that regions of the human mPFC including the vmPFC and the dmPFC share some homology with regions within the rodent mPFC (**Figure 1**). The rodent prelimbic (Riedel et al., 2009) cortex is considered homologous to BA 32, which lies within the mPFC and vmPFC (Wise, 2008), although some have suggested this region contains some similarities with human dlPFC as well (Uylings et al., 2003). The rodent infralimbic cortex (IL) is considered homologous to BA 25, and lies within the vmPFC (Wise, 2008). Additionally, the rodent mOFC is considered homologous with the human mOFC (Preuss, 1995). The human dmPFC includes parts of the dorsal ACC, which shares homology with the rodent ACC (Wise, 2008). Other regions of the human PFC are generally considered not to share homology with the rodent brain. Rodents do not have a granular PFC, and therefore granular regions of the human PFC including the dlPFC do not have a homologous structure within the rodent brain (Preuss, 1995; Wise, 2008; but see Uylings et al., 2003). In this review, we discuss the evidence that the PFC might regulate social behaviors in rodents, as well as in humans, and that pathologies leading to social deficits in rodent models of psychiatric disease might be related to altered functioning in the PFC. Although there are clear differences between social cognition in humans and rodents, there are common underlying functions that are achieved in species-specific ways. For this reason, it is important to use ethologically relevant behavioral models that capitalize on natural rodent behaviors requiring social processing (Thompson and Levitt, 2010). Here we review a burgeoning literature examining the PFC contribution to social behaviors in rodents. We focus on behaviors that are not directly related to mating or parent–offspring relationships but that are ethologically relevant to social processing demands in rodents, including social motivation/affiliation, social memory/recognition, and dominance. These behavioral domains can be conceptually compared to the human categories: social motivation, knowledge of self and others, and hierarchies within groups.

**FIGURE 2:**
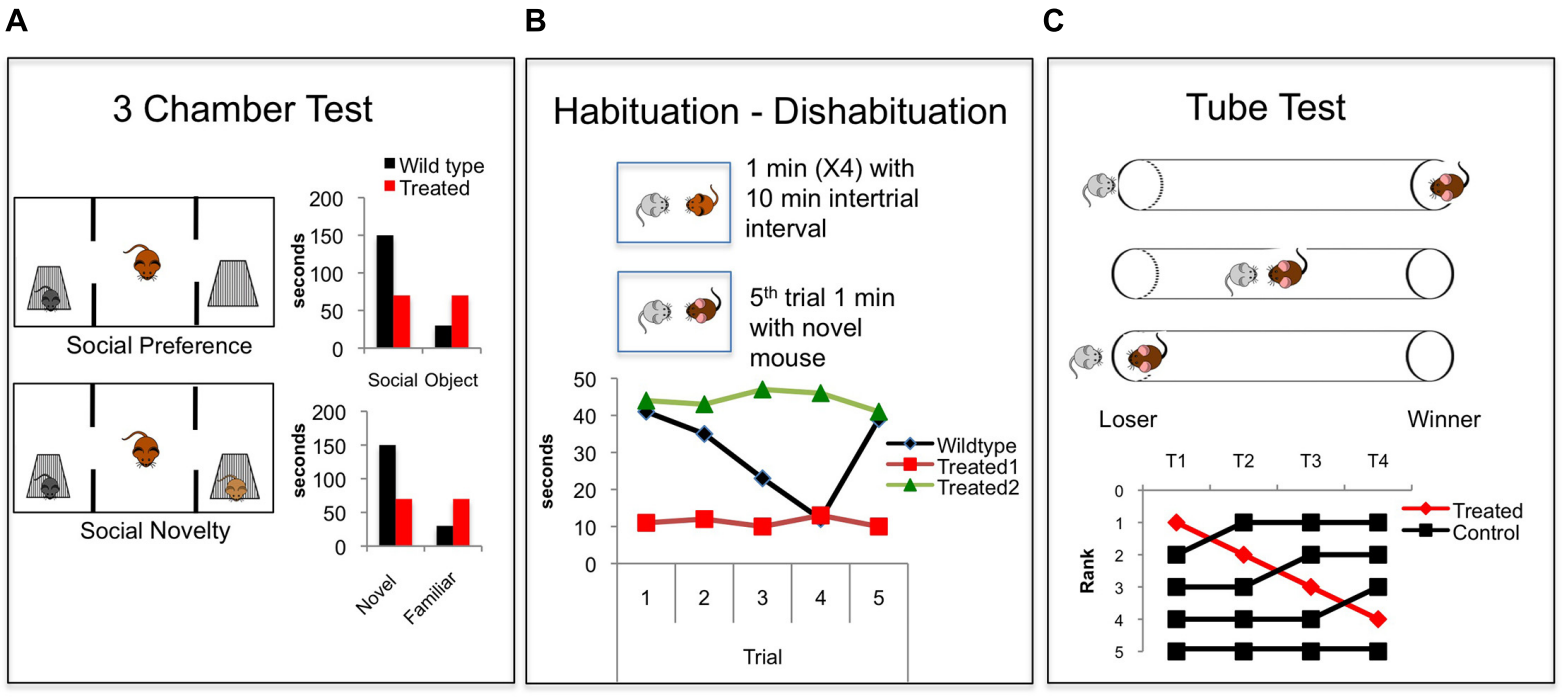
**Common behavioral paradigms for studying social cognition in rodents.** **(A)** The three chamber test (Moy et al., 2004). In the first phase social preference is assessed. A focal mouse chooses between a social target and an object and time spent investigating both is measured and compared. In the second phase social novelty preference is assessed when a novel mouse is added and the focal mouse chooses to investigate a novel vs. familiar mouse. Graphs show common findings demonstrating the natural wildtype (black bars) propensity to investigate a social target more than an object, and to investigate a novel mouse more than a familiar mouse. Red bars demonstrate a hypothetical treated group showing no social preference and no novel social preference. **(B)** The Habituation – Dishabituation paradigm (Thor and Holloway, 1982) in which a juvenile mouse is presented to a focal mouse, usually in the home cage, for four consecutive 1 min trials with an intertrial interval of 10 min. A novel juvenile is presented on the fifth trial. The graph shows commonly reported wildtype social investigation time (black), which decreases over the four trials and then increases with the presentation of the novel mouse on the fifth trial, demonstrating recognition of a novel animal. Hypothetical red data shows floor levels of social investigation, similar to that seen when animals are treated with NMDAR antagonists (Zou et al., 2008; Jeevakumar et al., 2015). Data indicated by the green line shows a ceiling level of social investigation showing hypothetical intact social motivation and decreased social recognition. This effect is seen in animals lacking the oxytocin gene (Ferguson et al., 2000). **(C)** The tube test. Tests for dominance by placing two mice into a tube and recording which mouse forces the other to back out of the tube (Lindzey et al., 1966). A fictitious experiment is shown in which the rank of the four control mice (black) is compared over time. The top ranked mouse is treated (red) and drops rank within the hierarchy. This effect is similar to that seen when the synaptic efficacy within the PFC is decreased (Wang et al., 2011a).

In rodents, behavioral paradigms that assess social motivation often rely on social preference tests that assess time spent with a novel social target compared with time spent with a novel object (Moy et al., 2004) (**Figure 2A**). These tests have frequently been used to assess social deficits in genetic mouse models of ASD (Silverman et al., 2010). The interaction typically takes place in a three chamber apparatus that allows for preference of the social chamber to be assessed. In these tests, the novel social stimulus is generally constrained in a compartment that allows sniffing and interaction but no physical contact. This controls for the behavior of the stimulus to influence the social interaction. Other paradigms simply measure the time spent investigating in an unconstrained interaction.

In mice and rats, paradigms aimed at measuring levels of social recognition exploit a natural propensity of mice to habituate to a familiar conspecific, and to explore a novel mouse more than a familiar one (Thor and Holloway, 1982) (**Figure 2B**). A focal mouse is exposed to a novel stimulus mouse, generally an ovariectomized female or a juvenile to diminish aggressive behavior. The presentation of the stimulus mouse is repeated multiple times with a delay between presentations. A decrease in the sniffing time across the repeated trials reflects recognition that the mouse is familiar. After repeated presentations, a novel mouse is presented, and increased investigation of the novel mouse reflects social novel preference (Thor and Holloway, 1982) (**Figure 2B**). This can also be assessed in the three chamber apparatus: After the social vs. object presentation, a second social target is added to the opposite chamber, and increased investigation of the novel vs. the familiar animal reflects a social novelty preference that relies on the recognition of a novel animal (Moy et al., 2004) (**Figure 2A**).

An interesting line of translational research aims to study empathy behavior in rodent models. This research generally follows one of two behavioral paradigms. The first capitalizes on the ability of mice and rats to alter their behavior by observing conspecifics (Choleris et al., 1997; Chen et al., 2009). For example, mice and rats demonstrate social transmission of pain (Langford et al., 2006), fear (Chen et al., 2009; Kim et al., 2012), and food preference (Choleris et al., 1997). The second general method assays prosocial behavior by placing rats in a situation where they have the opportunity to free a trapped conspecific in the presence of a valued food source (Bartal et al., 2011).These tests provide an interesting opportunity to examine changes in empathy behavior in animal models of ASD and SCZ.

A third dimension of social behavior in rodents is social hierarchy and dominance. In mice, social hierarchies develop when mice live in high-density conditions, and this likely allows for a decrease in aggressive behavior and an increase in social tolerance (Anderson, 1961). These social hierarchies can be assessed in several ways. A simple way is to observe behaviors of animals in their home cage, or to observe aggressive behavior interactions that typically happen when a group is placed into a new cage. Measurements of biting, attacks, and submissive postures can be used to infer dominance relationships in a group. New automatic systems used to track social dynamics of large groups of mice in complex environments have added to this body of work (Shemesh et al., 2013; Weissbrod et al., 2013). Another way to measure dominance is through a tube test method (Lindzey et al., 1966) (**Figure 2C**). In this paradigm, mice are placed in pairs, facing each other, into a tube that does not allow enough space for mice to pass each other or for either mouse to turn around. One mouse is forced to back out of the tube (‘loser’) by the other mouse (‘winner’). This test allows for the inference of dominance relationships between pairs of mice, and is very highly correlated with other measures of dominance including marking in a novel environment and vocalizations in a mating context (Wang et al., 2011a, 2014).

In the following, we review evidence that rodent social behavior including social motivation, recognition of conspecifics, empathy behavior, and hierarchy are altered by activity within the rodent PFC.

## Social Motivation And PFC In Rodents

### PFC Regulation of Social Motivation in Rodents

Social motivation describes the motivation of an animal to approach, explore, and otherwise interact with a social target. Social motivation is disrupted in ASD (Chevallier et al., 2012) and SCZ (Fervaha et al., 2015). Research from animal models supports the human literature implicating the contribution of the PFC in social motivation, in conjunction with subcortical areas that mediate rewarding aspects of social interaction like the NAc and VTA (Gunaydin et al., 2014; Kas et al., 2014). Lesions of regions within the rodent PFC have demonstrated its’ importance in social functioning. For example, lesioning the rat OFC disrupts play behavior and increases aggressive behavior (Pellis et al., 2006; Rudebeck et al., 2007). Lesions of the ACC disrupt social memory and decrease social interest (Rudebeck et al., 2007) and lesions of the PL region of the rodent PFC actually increase social investigation, possibly due to an increase in aggression (Avale et al., 2011). Therefore, lesion studies have provided evidence for the necessary role of the PFC in social motivation, however, lesions are a crude manipulation that often damages adjacent regions and passing axons. A recent study examined whole brain cfos activity in a social context and found that the mouse PFC was activated in a social interaction (Kim et al., 2015), showing a correlative involvement of the PFC in social behavior.

**FIGURE 3:**
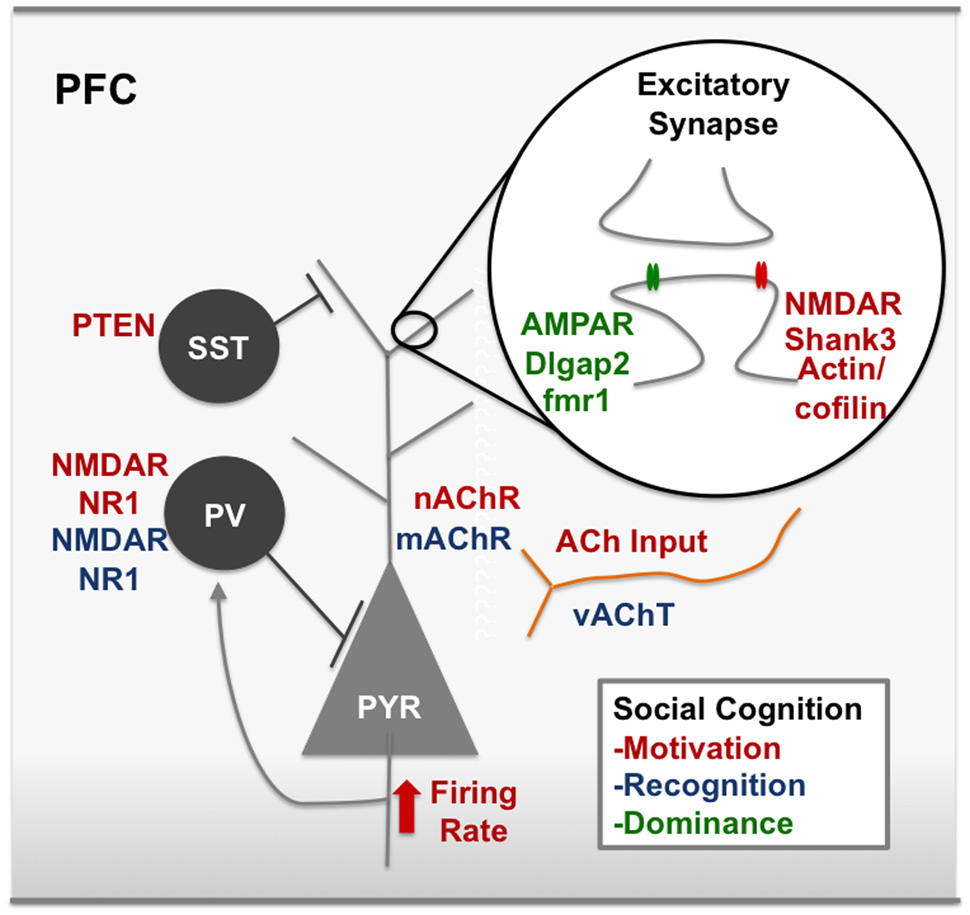
**Modulators of social cognition in the rodent PFC.** Red text represents nodes of the circuit that, when disrupted, decrease social motivation. For example, synaptic scaffolding proteins on excitatory synapses like Shank3 and IRSp53 have been associated with social motivation in the PFC, as have cytoskeleton remodelers, actin and cofilin. NMDARs at excitatory synapses are also a key node of the social motivation circuit. ACh input to the PFC and nicotinic receptors have also been shown to modulate social motivation, however, it is unclear which cell types are important for ACh action or whether these effects are pre or post-synaptic. Blue text represents nodes of the circuit that, when disrupted, decrease social recognition. For example, disrupting gabaergic neurotransmission by removing the NR1 subunit on cortical gabaergic interneurons disrupts social recognition. Green text represents nodes of the circuit that are involved in dominance behavior. For example, bidirectional modulation of AMPARs and mutations in the fmr1 gene. ACh, acetylcholine; AMPAR, α-amino-3-hydroxy-5-methyl-4-isoxazolepropionic acid receptor; dlgap2, disks large-associated protein 2, fmr1, fragile X mental retardation 1; IRSp53, insulin receptor substrate protein of 53 kDa, mAchR, muscarinic acetylcholine receptor, nAChR, nicotinic acetylcholine receptor, NMDAR, *N*-Methyl-D-aspartate receptor, PV, parvalbumin postitive interneuron, vAChT, vesicular acetylcholine transporter. See text for references.

The microcircuitry of the PFC contains a complex array of interneurons that inhibit circuit activity, as well as neuromodulator inputs including acetylcholine (ACh), dopamine (DA), and oxytocin (OT). The concept of Excitatory/Inhibitory balance (E/I balance) is a broad term that attempts to capture alterations within the circuit that alter the ratio of excitatory:inhibitory neurotransmission. The influence of changing E/I balance in the developing cortex has been extensively linked to critical period plasticity (Hensch, 2003, 2004, 2005). Interestingly, decreases in inhibitory neurotransmission are a common finding in animal models of ASD (Gogolla et al., 2009). Many human studies of ASD (Rubenstein and Merzenich, 2003) and SCZ (Sun et al., 2013) patients also show decreases in inhibitory neurotransmission measured by decreased power of gamma oscillations, an indication of decreased activity of fast-spiking inhibitory basket cells (Bartos et al., 2007). Human post mortem studies of ASD patients have shown increases in dendritic spines in cortical regions, and overall increased within-region interconnectivity and decreased long-range interconnectivity, particularly in the frontal cortex (Wass, 2011). Post mortem studies of SCZ PFC has shown decreased markers of inhibitory neurons (Akbarian et al., 1995; Mitchell et al., 2015). Examining how alterations in the balance of circuits within the PFC alters social motivation is crucial to identifying underlying pathology of social deficits. In the following we review evidence that alterations within the microcircuitry of the PFC interfere with social motivation, and provide a framework for understanding human psychiatric diseases with social deficits (**Figure 3**).

Direct alterations of E/I balance within the PFC in adult mice have a strong effect on social motivation. For example, Yizhar et al. (2011) used optogenetics to independently manipulate the activity of excitatory pyramidal neurons and inhibitory parvalbumin (PV) interneurons within the PFC both during a social exploration task, and in the three chamber sociability test (**Figure 3**) (Yizhar et al., 2011). They found elevating the excitatory balance by stimulating pyramidal neurons in the PFC abolished social exploration and disrupted social preference in the three chamber test. On the other hand, there was no effect on social motivation when inhibition was increased by stimulating PV interneurons. The effects of increased excitation were ameliorated by simultaneously stimulating PV interneurons, showing that an appropriate E/I ratio in the PFC is required for social motivation in mice. These findings corroborate human literature that shows a role for altered E/I balance within the PFC in psychiatric disorders including SCZ and ASD (Toro et al., 2010; Morishita et al., 2015).

### Neuromodulators in the PFC Modulate Social Motivation

In addition to direct alterations in glutamatergic and gabaergic neurotransmission, many neuromodulators alter microcircuitry activity. The neuromodulator acetylcholine (ACh) acting within the cortex modulates social motivation, since selective denervation of cholinergic input to the neocortex in rats significantly reduces social motivation (Savage et al., 2011). However, in mice lacking the ß2 nicotinic receptor there is actually an increase in social contact, which is normalized with virally mediated ß2 rescue within the PL region of the PFC. This suggests that ACh signaling through nicotinic receptors in the PL may actually attenuate social motivation, perhaps in favor of novel context exploration (Avale et al., 2011) (**Figure 3**). Other neuromodulators, like neuropeptides may alter social motivation through signaling in the PFC. OT has been shown to mediate many pair bonding and social affiliative mechanisms, but much of this work has examined OT within subcortical structures (Insel and Young, 2000). New research examining the role of OT in the cortex has shown that OT mediates the salience of pup calls through modulating E/I balance in the auditory cortex of dams (Marlin et al., 2015), and modulates cross modal experience-dependent plasticity between multiple sensory cortices (Zheng et al., 2014). Additionally, there is a population of SST interneurons in the mPFC that express the OT receptor and have differential responses to OT in male and female mice (Nakajima et al., 2014). These OTR expressing neurons in the mPFC specifically regulate the social motivation of female mice to interact with male mice during estrus, without affecting the motivation to interact with another female mouse. Therefore OT acting in conjunction with steroid hormones could play a key role in modulating other aspects of social cognition through actions in the mPFC. More research delving into the effects of neuropeptides in the cortex will elucidate mechanisms by which these modulators may affect E/I balance within the PFC and social motivation.

### Cortical E/I Balance and Social Motivation: Relevance to Genetic Animal Models of ASD and SCZ

Other studies have examined E/I balance in the context of genetic risk factors for ASD and SCZ, and found alterations in E/I balance within the cortex in general, and in some cases in the PFC specifically, in animal models showing decreased social motivation (**Figure 3**). For example, transgenic mice expressing only ∼10% of normal levels of the NR1 subunit of the *N*-Methyl-D-Aspartate (NMDA) receptor show decreased social motivation, decreased ultra sonic vocalizations (USVs), and abnormal gamma synchrony (Gandal et al., 2012a). This study also demonstrated that this model of hypofunction of NMDARs increased E/I ratio, specifically pyramidal neuron excitability (Gandal et al., 2012b). This model is relevant to both ASD and SCZ, as it models overlapping symptoms of the social motivation deficits and the abnormal cortical E/I balance. Mice lacking the expression of PV, a calcium binding protein that defines a population of interneurons, show a constellation of ASD behavioral phenotypes, including decreased social interaction (Wohr et al., 2015). The loss of this calcium binding protein also increases inhibition within the cortex. Therefore, decreasing E/I ratio in the cortex, as well as increasing it, interferes with normal social motivation. These findings can be interpreted in the context of SCZ as well as ASD, since SCZ patients show PV interneuron dysfunction in post mortem brain (Lewis et al., 2005). The phosphatase PTEN has also been implicated in ASD (Butler et al., 2005), and in a recent paper, Vogt et al. (2015) used a conditional knockout strategy targeted to the medial ganglionic eminence to remove PTEN in interneuron progenitors. Using this strategy, they observed an overall loss of interneurons and a preferential loss of somatostatin (SST) positive interneurons compared with PV neurons in cortex, hippocampus, and striatum. Surprisingly, the loss of these inhibitory interneurons actually resulted in an increase in inhibition onto layer 2/3 neocortical pyramidal neurons, decreased social motivation, and increased gamma oscillations during social interaction compared with controls. Genetic ablation of the NR1 subunit of NMDARs on PV interneurons causes alterations in mouse electroencephalograph (EEG) recordings in response to an auditory stimulus, a finding seen in Autistic patients (Roberts et al., 2010). These mice also show social motivation deficits and reduced USVs in a mating context (Saunders et al., 2013). Many genetic models of ASD and SCZ that result in social deficits are caused by loss of function of synaptic adhesion molecules or scaffolding proteins in the PSD. For example, mice with deletions or mutations in the cell adhesion proteins Neuroligin-1 (NL-1) (Blundell et al., 2010), Neuroligin-3 (NL-3) (Tabuchi et al., 2007), and Neuroligin-4 (NL-4) (Jamain et al., 2008) show social motivation deficits. Alterations in neuroligins have widespread effects on excitatory/inhibitory balance throughout the brain (Maćkowiak et al., 2014). Additionally, mice with mutations in the ASD- associated post-synaptic density (PSD) protein Shank3 (Wang et al., 2011b; Betancur and Buxbaum, 2013) show social motivation deficits and altered E/I balance (Lee et al., 2015). Findings from these studies show that an altered E/I ratio, most often caused by disruptions in inhibitory neurotransmission can lead to social motivation deficits.

While many of the genetic contributions to social motivation deficits alter global E/I balance, some of these deficits have shown specific PFC related deficiencies. One study examining Shank3 deficient mice, found that decreased social motivation in this genetic model is specific to the PFC. Shank3 deficient mice show decreased NMDA mediated excitatory post-synaptic current (EPSC) amplitude in layer 5 pyramidal neurons as well as a decrease in F-actin filaments within this region. The social deficits, as well as the decreases in NMDAR expression and function, can be rescued by inhibiting the main actin depolymerizing factor, cofilin, either systemically or specifically in the PFC (Duffney et al., 2015). Additionally, inhibiting NMDARs in the PFC is sufficient to reproduce the loss of social motivation. This study suggests that an intact actin cytoskeleton is required for normal excitatory transmission through NMDARs, and that these components are required in the PFC for intact social motivation (**Figure 3**). Another study examined the loss of a different excitatory PSD protein, the insulin receptor substrate protein, (IRSp53) and found that mice lacking this gene show decreased social motivation and reduced excitatory neurotransmission in layer 2/3 of the mPFC as well as decreases in dendritic spine number and maturity in this region. The social deficits, as well as the decreased excitatory neuronal firing rate in the mPFC, are rescued by normalizing the altered E/I balance with an NMDAR antagonist (Chung et al., 2015) (**Figure 3**). Mice lacking the methyl CpG binding protein 2 (MeCP2) that is known to cause Rett syndrome (Amir et al., 1999), show social avoidance (Moretti et al., 2005) and a mPFC specific dysfunction of excitatory neurotransmission (Sceniak et al., 2015). These findings taken together suggest that altered social motivation in animal models of ASD is linked to alterations in the E/I balance that is in some cases specifically relates to the microcircuitry of the PFC. In contradiction to this theory, causing NMDAR dysfunction specifically in adulthood in the mPFC of mice with a cre-mediated excision of the NR1 subunit did not decrease either social preference or social novelty preference in the three chamber test (Finlay et al., 2015). This finding points to the importance of studying how circuits within the PFC develop, as supposed to their functioning in adulthood (Ueda et al., 2015).

## Social Recognition And PFC In Rodents

### Does the PFC regulate Social Recognition in Rodents?

Social recognition and memory are key aspects of social cognition and normal social functioning, and are considered requirements for forming long-term attachments, hierarchies, and other complex social strategies in animals. In humans, social recognition is one component of knowledge of self and others, and is an important prerequisite for other forms of social cognition including empathy and moral decision-making. Early social recognition is also disrupted in children with ASD, and performance on a facial recognition task predicts future symptom severity (Dawson et al., 2002a; Eussen et al., 2015).

Circuits involved in social recognition in rodents depend in part on the hippocampus and medial amygdala (MeA), perhaps not surprisingly given the importance of the hippocampus for memory formation (Kogan et al., 2000), and the MeA in processing volatile scent cues (Noack et al., 2015). In mice, the volatile fraction of the scent cue is required for recognition memory (Noack et al., 2010) and retrieval of these cues depends on the MeA (Noack et al., 2015). The neuropeptides oxytocin (OT) and vasopressin (AVP) are also part of a canonical social recognition circuit and signaling of these peptides through the MeA and Lateral Septum (LS), respectively, is required for social recognition. (Ferguson et al., 2000, 2001; Bielsky et al., 2004, 2005a,b). There is some evidence that the PFC is involved in social recognition. For example, one study showed that lesioning the ACC, but not the OFC disrupts social recognition in rats (Rudebeck et al., 2007). Fibroblast growth factor 17 (Fgf17) is a secreted signaling molecule involved in patterning the development of the rostral forebrain (Cholfin and Rubenstein, 2007). Fgf17 deficient mice show deficits in social recognition and decreased activation of the immediate early gene, Fos, in the frontal cortex after exploring a novel environment with an opposite sex partner (Scearce-Levie et al., 2008). This evidence points to the importance of the frontal cortex in social recognition (Scearce-Levie et al., 2008) and the ACC specifically in rats (Rudebeck et al., 2007), however, evidence for a role of the PFC in social recognition in rodents is not yet conclusive.

### E/I balance and Social Recognition: Translational Relevance

An influential hypothesis links SCZ pathology, and in particular the negative symptoms of this disease, including defects in social cognition, to hypofunction of the NMDA receptors on inhibitory interneurons (Kehrer et al., 2008). Non-competitive NMDA receptor antagonists like MK801 or ketamine preferentially decrease activity of inhibitory interneurons within the cortex, thereby increasing glutamatergic tone and E/I ratio through disinhibition (Moghaddam et al., 1997; Homayoun and Moghaddam, 2007; Gordon, 2010). Pharmacologically disrupting E/I ratio with these agents also produces a variety of schizophrenia-like behaviors in animal models (Moghaddam et al., 1997; Homayoun and Moghaddam, 2007). Tests of the NMDAR hypothesis of SCZ have revealed the importance of NMDAR functioning and intact E/I balance in social recognition, yet no study has pointed to disrupted E/I balance specifically within the PFC as causally disrupting social recognition. Acute injections of MK801 decrease social exploration of a novel juvenile and decrease social recognition (Zou et al., 2008) and postnatal ablation of the NR1 subunit of the NMDA receptor in 40–50% of γ –Aminobutyric acid (gabaergic) interneurons in the cortex and hippocampus abolishes short-term social memory, without affecting overall levels of social investigation (Belforte et al., 2010). Both of these studies suggest loss of inhibitory tone decreases social recognition. When ketamine is given during the second postnatal week of development it preferentially decreases PV expression in the mPFC, decreases GABA release in layers 2/3, and increases spontaneous glutamatergic inputs onto PV cells, consistent with an increase in the E/I balance within the cortex (Jeevakumar and Kroener, 2014). This treatment decreases social exploration and disrupts social recognition (Jeevakumar et al., 2015). Collectively, these studies suggest that increased E/I ratio in the cortex caused by NMDAR hypofunction on inhibitory neurons leads to deficits in social recognition (**Figure 3**). Retention of social memories is enhanced by activating NMDARs (Hlinak and Krejci, 2002), showing a bidirectional modulation of social recognition by NMDAR activity. Taken together these findings demonstrate the robust importance of NMDARs and intact E/I balance for social memory. While most of these studies aim to investigate animal models of the glutamate hypothesis of SCZ, these findings are also likely to be relevant for ASD, since human studies have shown disinhibition and decreased inhibitory functioning in humans with both ASD (Rubenstein and Merzenich, 2003) and SCZ (Uhlhaas and Singer, 2015). However, future studies are required to determine which specific regions within the cortex require NMDAR mediated responses in order to perform normal social recognition.

### Neuromodulators in the PFC Modulate Social Recognition

Some evidence for a PFC contribution to normal social recognition comes from studies examining the pharmacology of social recognition in rats. These studies have outlined the importance of the neuromodulators acetylcholine (ACh) and dopamine (DA) within the frontal cortex for normal social memory (**Figure 3**). The muscarinic receptor antagonist, scopolamine, decreases short-term social memory in the three chamber test in mice without affecting social preference (Riedel et al., 2009). In rats, a scopolamine-induced social recognition deficit is attenuated by administering a nicotinic receptor agonist (Van Kampen et al., 2004). Other findings have suggested the reduced social recognition seen after scopolamine injection may be mediated through the melanin-concentrating hormone (MCH) receptor, since in a separate study the effect of scopolamine was dose-dependently blocked using an MCH receptor blocker (Millan et al., 2008). MCH and ACh neurotransmission interact in the Frontal Cortex to produce effective social recognition, since MCH receptor blocking elevates extracellular dialysates of ACh in the PFC and enhances social recognition (Millan et al., 2008). Dopamine signaling also modulates ACh levels in the PFC and social recognition: Administration of a dopamine (D3) receptor antagonist creates an elevation of ACh specifically in the PFC, and attenuates the negative effects of scopolamine on social memory in rats (Millan et al., 2007). Additionally, mice with a heterozygous deletion of the acetylcholine transporter VAChT show impaired object and social recognition (Prado et al., 2006). This finding suggests that general habituation-related memory may be impaired after decreasing vesicular trafficking of ACh and not social recognition memory specifically. In fact, many studies examining social recognition deficits see broad memory effects and not social recognition deficits specifically. Therefore, ACh neuromodulation in the PFC may affect social recognition through ‘domain general’ mechanisms like attention or working memory.

### Does the PFC Regulate Empathy Behaviors in Rodents?

Learning by observing conspecifics provides a strong evolutionary advantage to social species. Observational learning and emotional contagion have been put forth as the evolutionary basis of empathy (Olsson et al., 2007). Interestingly, this type of learning engages brain regions within the mPFC, including the ACC in both humans (Singer et al., 2004; Olsson et al., 2007) and rodents (Jeon et al., 2010; Jurado-Parras et al., 2012; Kim et al., 2012). For example, mice acquire a conditioned contextual fear by observing conspecifics, and this behavior is dependent on the right ACC (Kim et al., 2012). Mice also learn more quickly to lever press for food if they observe a well-trained demonstrator, and this advantage is abolished if the mPFC is electrically stimulated during the observational learning (Jurado-Parras et al., 2012). Injection of the antipsychotic haloperidol or serotonin into the ACC of mice in an observational fear-learning task decreased the expression of conditioned fear (Kim et al., 2014). Serotonin microinjection in this study reduced gamma-band activity in this region, suggesting serotonin modulation of ACC activity disrupts social learning. These results clearly implicate the ACC in empathy related behaviors in mice.

## PFC Regulation Of Social Hierarchy In Rodents

Social hierarchies are common among mammals and likely confer an important adaptation to living in groups (Cummins, 2000). In humans and non-human primates a dominance hierarchy involves recognizing dominance relationships, learning social norms, and reading intentions of others (Cummins, 2000). For this reason, hierarchy represents a complex form of social cognition that requires plasticity of behavior in the face of changing social contexts. In mice, dominance also seems to be linked to the microcircuitry in the PFC (Wang et al., 2014). For example, altering the efficacy of synaptic transmission in the PFC causes a bidirectional modulation of social hierarchy (Wang et al., 2011a) (**Figure 3**). Specifically, increasing excitability using a viral strategy that increases AMPA receptor trafficking to the synapse increases the mouse’s rank within the hierarchy. Conversely, dampening the efficacy of synaptic transmission by decreasing AMPARs at the synapse decreases the rank. This study also found an increase in the amplitude of EPSCs in dominant compared with subordinate mice. Additionally, dominance behavior studies in mice have been useful in animal models of social cognition deficits in ASD. For example, knocking out Dlgap2, an important PSD scaffolding protein associated with ASD, increases dominance and aggressive behavior and decreases AMPAR-mEPSCs and spine density in the mouse OFC (Jiang-Xie et al., 2014). Optogenetically activating the mPFC (PL/IL) in mice decreases aggressive behavior, while silencing this region leads to an escalation of aggression (Takahashi et al., 2014). This finding is interesting in light of the findings of Wang et al., because these studies together demonstrate opposing regulation of aggression and dominance by activity of the mouse PFC (Wang et al., 2011a). In conclusion, social hierarchy is modulated by excitatory neurotransmission in the PFC, and is a useful way to investigate social cognition alterations in genetic animal models of human disease.

## Conclusion

Our knowledge and understanding of the neural mechanisms governing social cognition is rapidly expanding, and a growing body of evidence points to the PFC as a central regulator. Social cognition involves integrating many behavioral domains including motivation and reward, salience, attention, flexibility, and a host of other processes. Not surprisingly, social cognition is affected in a wide variety of psychiatric disorders. We have reviewed evidence that alterations in the microcircuitry of the PFC are related to social motivation deficits in animal models of ASD and SCZ. Models of social memory have pointed to the importance of the neuromodulators acetylcholine and dopamine within in the PFC. Additionally, the glutamate hypothesis of SCZ has led to an understanding of the requirements of NMDA receptor functioning and E/I balance in social recognition. Studies of social hierarchy point to a causal role of synaptic efficacy within the PFC in mediating dominance in mice (Wang et al., 2011a). These studies are supported by human literature, which implicates the PFC in studies of social cognition including motivation, knowledge of self and others, and social structures. Taken together, the evidence suggests that the PFC is a hub that regulates multiple components of social cognition across species. We predict that future exploration of prefrontal microcircuitry in rodent models will provide novel insights into the deficits in the social domain frequently associated with psychiatric disorders (**Table 2**).

**Table 2:** Questions for future research.

This review presented evidence that the PFC is a common regulator across social behaviors in rodents, and that E/I balance, specifically within the PFC effects social cognition. However, many outstanding questions remain:
• How does the development of circuits within the PFC contribute to the development of social cognition? In humans, social cognition has a clear developmental trajectory, but the extent of this development is still unclear in animal models. Answering questions about the ways in which maturation of PFC circuits leads to appropriate development of social cognition in animals will improve our understanding of neurodevelopmental diseases like Autism and Schizophrenia.
• What are the properties of the regulation of E/I balance during development and how does E/I balance over the course of development contribute to normal social functioning in adulthood?
• How do different cell types and microcircuits within the rodent PFC contribute to E/I balance development and social behavior?
What are the circuits that connect the mPFC to other regions of the ‘social brain’ and how are distinct social behaviors regulated by these circuits?
• What is the role of E/I balance within the PFC in social recognition? While lots of evidence points to NMDAR functioning and E/I balance as necessary for social recognition, no study has specifically tested the causal relationship between E/I balance in the PFC and social memory.
• Are there sex differences in these behaviors? Most of the research on social behavior comes from male mice, and so while we know female mice also show social motivation, social recognition, and social hierarchy, we don’t know whether there are sex differences in the neural mechanisms underlying these behaviors.


## Conflict of Interest Statement

The authors declare that the research was conducted in the absence of any commercial or financial relationships that could be construed as a potential conflict of interest.

## Footnotes

1 http://www.nimh.nih.gov/research-priorities/rdoc/social-processes-workshop-proceedings.shtml
